# Incidence, risk factors, and prognostic indicators of symptomatic air embolism after percutaneous transthoracic lung biopsy: a systematic review and pooled analysis

**DOI:** 10.1007/s00330-020-07372-w

**Published:** 2020-10-13

**Authors:** Jong Hyuk Lee, Soon Ho Yoon, Hyunsook Hong, Ji Young Rho, Jin Mo Goo

**Affiliations:** 1 Department of Radiology, Seoul National University Hospital, Seoul National College of Medicine, Seoul, South Korea; 2 Medical Research Collaborating Center, Seoul National University Hospital, Seoul, South Korea; 3 Wonkwang University Hospital, Wonkwang University College of Medicine, Iksan, South Korea

**Keywords:** Radiology, interventional, Embolism, air, Biopsy, Lung

## Abstract

**Objectives:**

To determine the incidence, risk factors, and prognostic indicators of symptomatic air embolism after percutaneous transthoracic lung biopsy (PTLB) by conducting a systematic review and pooled analysis.

**Methods:**

We searched the EMBASE and OVID-MEDLINE databases to identify studies that dealt with air embolism after PTLB and had extractable outcomes. The incidence of air embolism was pooled using a random effects model, and the causes of heterogeneity were investigated. To analyze risk factors for symptomatic embolism and unfavorable outcomes, multivariate logistic regression analysis was performed.

**Results:**

The pooled incidence of symptomatic air embolism after PTLB was 0.08% (95% confidence interval [CI], 0.048–0.128%; *I*^2^ = 45%). In the subgroup analysis and meta-regression, guidance modality and study size were found to explain the heterogeneity. Of the patients with symptomatic air embolism, 32.7% had unfavorable outcomes. The presence of an underlying disease (odds ratio [OR], 5.939; 95% CI, 1.029–34.279; *p* = 0.046), the use of a ≥ 19-gauge needle (OR, 10.046; 95% CI, 1.103–91.469; *p* = 0.041), and coronary or intracranial air embolism (OR, 19.871; 95% CI, 2.725–14.925; *p* = 0.003) were independent risk factors for symptomatic embolism. Unfavorable outcomes were independently associated with the use of aspiration biopsy rather than core biopsy (OR, 3.302; 95% CI, 1.149–9.492; *p* = 0.027) and location of the air embolism in the coronary arteries or intracranial spaces (OR = 5.173; 95% CI = 1.309–20.447; *p* = 0.019).

**Conclusion:**

The pooled incidence of symptomatic air embolism after PTLB was 0.08%, and one-third of cases had sequelae or died. Identifying whether coronary or intracranial emboli exist is crucial in suspected cases of air embolism after PTLB.

**Key Points:**

• *The pooled incidence of symptomatic air embolism after percutaneous transthoracic lung biopsy was 0.08%, and one-third of patients with symptomatic air embolism had sequelae or died.*

• *The risk factors for symptomatic air embolism were the presence of an underlying disease, the use of a ≥ 19-gauge needle, and coronary or intracranial air embolism.*

• *Sequelae and death in patients with symptomatic air embolism were associated with the use of aspiration biopsy and coronary or intracranial locations of the air embolism.*

## Introduction

Percutaneous transthoracic lung biopsy (PTLB) is a well-established image-guided procedure for the diagnosis of lung parenchymal lesions [1, 2] with excellent diagnostic accuracy [3, 4]. Nevertheless, PTLB can be accompanied by complications, including pneumothorax (9 to 54% with an average of around 20%), lung parenchymal hemorrhage (11%), and hemoptysis (5.2 to 7%) [5–7]. The majority of these complications can be managed conservatively or with a minimal intervention, such as percutaneous tube drainage, without any sequelae [5, 6, 8].

Air embolism is another complication of PTLB [6–11]. It occurs extremely rarely [12–14], but can result in mortality or sequelae [15–17], and therefore constitutes a significant concern for radiologists. However, recent studies have reported cases with symptomatic air embolism after PTLB that resolved without sequelae [6, 8, 18, 19]. Due to the rarity of air embolism, most of the relevant publications reported a single case or a few cases, hampering a comprehensive understanding of air embolism after PTLB. A systematic review, followed by a pooled analysis, is a way to synthesize information from all the relevant publications regarding air embolism after PTLB. Thus, we conducted a systematic review and pooled analysis to determine the incidence, risk factors, and prognostic indicators of symptomatic air embolism after PTLB.

## Materials and methods

This systematic review was performed and reported in compliance with the Preferred Reporting Items for Systematic Reviews and Meta-Analyses (PRISMA) guidelines. The protocol of this study was registered in the International Prospective Register of Systematic Reviews (ID: CRD42019141479).

### Search strategy

We searched the EMBASE and OVID-MEDLINE databases to identify relevant publications with the following search terms: [(lung OR pulmonary) AND {(percutaneous OR transthora*) OR (biops* OR aspira* OR needle)}] AND {(air AND embol*) OR (gas AND embol*)}. The initial search was undertaken on May 8, 2019, and updated on January 5, 2020. We only included articles published in English without any limits on publication year.

### Eligible criteria for study selection

We reviewed all the searched publications regardless of the study type. The inclusion criteria were as follows: (a) studies with one or more patients suspected of having air embolism after PTLB, (b) confirmation of the presence of air embolism by autopsy or computed tomography (CT), and (c) sufficient data described in detail to extract the final outcomes and the variables associated with air embolism. We applied the following exclusion criteria: (a) studies with individuals who underwent bronchoscopic lung biopsy, not PTLB, and (b) individuals who underwent percutaneous needle localization, rather than biopsy.

We only included case reports, case series, prospective or retrospective cohort studies, and case-control studies, not letters, editorial comments, abstracts, review articles, guidelines, or consensus statements. Full-text articles were assessed for eligibility independently by two authors (J.H.L. and S.H.Y. with 7 and 14 years of experience in thoracic radiology, respectively). Any discrepancy was resolved by consensus.

### Data extraction

For data extraction, the two authors (J.H.L. and S.H.Y.) independently reviewed all eligible articles and extracted the following data using a standardized spreadsheet in Microsoft Excel 2016 (Microsoft Corporation): study characteristics, population characteristics, radiological characteristics of the biopsied lesion, information related to PTLB, information related to air embolism, and prognosis. In addition to this review, we tried to contact the corresponding authors of each study via e-mail for missing or unreported data.

### Methodological quality assessment of individual studies

Quality assessment was performed according to the tool proposed by Murad et al, which is composed of eight items categorized into four domains [20]. Three items in the causality domain were not adopted because they are mostly relevant to cases of adverse drug events [20]. We assessed the methodological quality of individual studies based on the five remaining items according to each purpose, which dealt with selection, ascertainment of exposure, causality, and reporting [20].

### Data synthesis and statistical analysis

We estimated the pooled incidence using a random-intercept logistic regression model, which is preferred over conventional approximate methods, especially for rare events [21]. Heterogeneity across the included studies was evaluated using the *I*^2^ statistic, which was derived from the Cochran *Q* statistic using the following equation, *I*^2^ = 100% · (*Q*−df)/*Q*. An *I*^2^ statistic > 50% was regarded as indicating substantial heterogeneity. A subgroup analysis and meta-regression were performed to investigate the causes of heterogeneity with the following covariates: guidance modality (CT guidance versus other modalities), study size (≥ 2500 vs. < 2500), and publication year (before 2000 vs. after 2000). The potential for publication bias was evaluated through a visual assessment of funnel plots of sample size against incidence due to the sparse event data [22].

We extracted individual-level data on patients with symptomatic or asymptomatic embolism from the included publications and analyzed the risk factors and prognostic indicators of symptomatic air embolism after PTLB. Unfavorable outcomes were defined as (a) patients’ survival with sequelae or (b) death. Since it was obvious that asymptomatic patients with air embolism after PTLB had favorable outcomes, we only included symptomatic patients in the analysis of unfavorable outcomes. The symptoms and locations of air embolism were categorized according to whether the brain or heart was involved because these organs play a critical role in patients’ outcomes [15–17]. For the univariate analysis, the Student *t* test for continuous variables and the Pearson chi-square test or Fisher exact test for categorical variables were performed. Subsequently, multivariate logistic regression analysis with backward stepwise selection was performed to identify predictors of symptomatic air embolism and unfavorable outcomes, respectively, using variables with a *p* value < 0.1 in the univariate analysis. Backward stepwise selection was conducted with an iterative entry of variables based on the test results (*p* < 0.05), and the removal of variables was based on likelihood ratio statistics with a probability of 0.10.

A *p* value of < 0.05 was considered to indicate a statistically significant difference, and statistical analyses were performed using SPSS version 21.0 (IBM Corp.) and R version 3.6.1 (R Foundation for Statistical Computing).

## Results

### Eligible studies and methodological quality

**Fig. 1:**
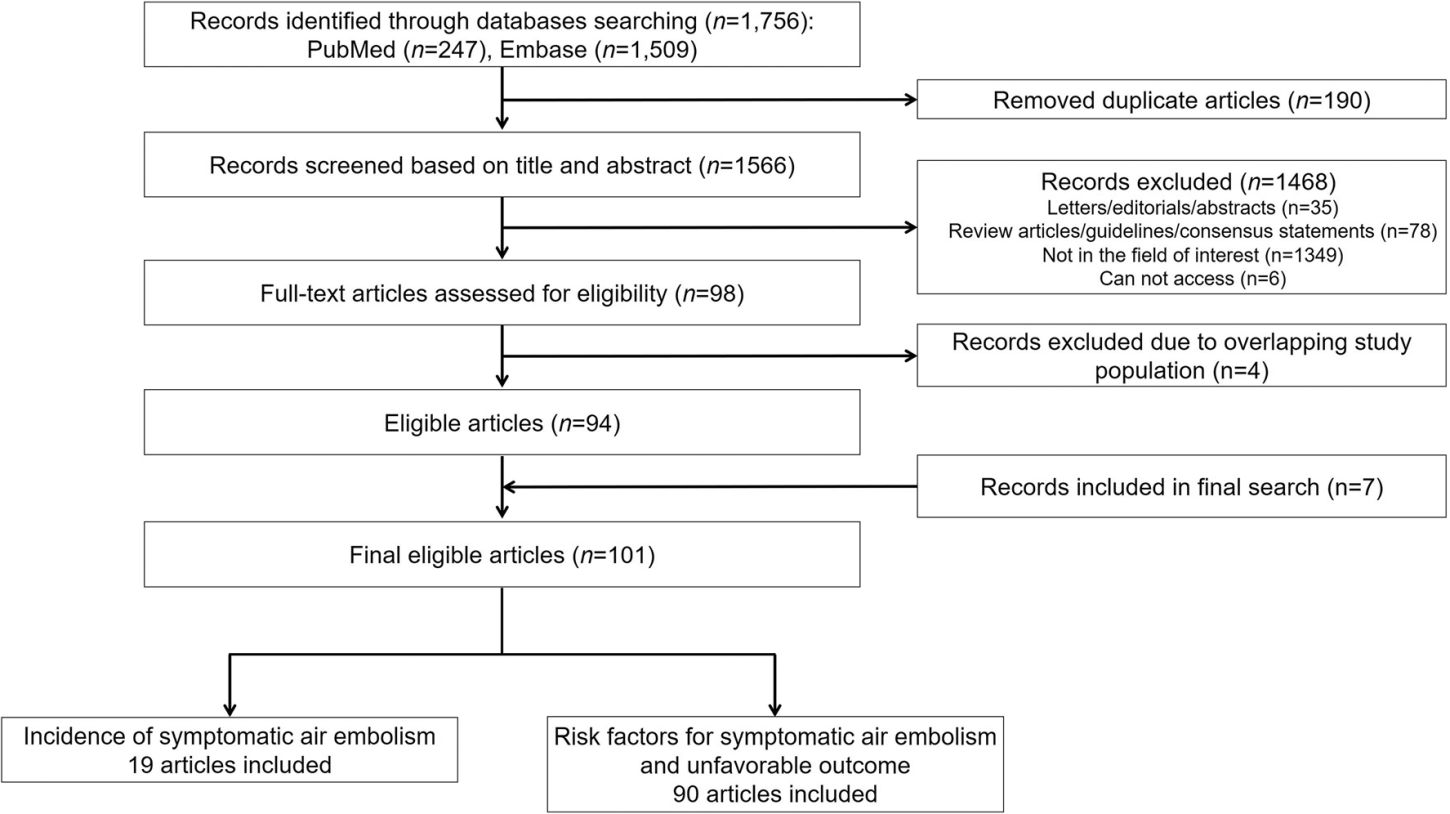
Flow diagram of the literature search

The initial literature search identified 1756 studies, of which 94 were eligible for the analysis of the incidence of symptomatic air embolism after PTLB or risk factors for symptomatic embolism and unfavorable outcomes. A supplemental search of the bibliographies of the retrieved publications added 7 studies. Finally, 101 studies were included in our analysis (Fig. 1).

In the methodological quality assessment, 19 articles with information on the incidence of symptomatic air embolism [3, 5, 6, 9, 10, 14, 18, 19, 23–33] and 90 articles with information on the risk factors for symptomatic air embolism and unfavorable outcomes were evaluated [6, 9, 14–19, 24, 26, 29, 34–112], respectively. For the incidence of symptomatic air embolism, all 19 articles were considered high quality, meeting all four items adequately. In the evaluation of risk factors, 81 of 90 articles were judged as high quality, as they satisfied all four items. However, seven case reports did not establish a causality between air embolism and PTLB because the air embolism occurred after repositioning of the biopsy needle to target the lung lesions before firing the biopsy needle [16, 24, 39, 53, 61, 89, 106]. Another case report was judged to have insufficient establishment of causality because it described a patient with air embolism just after removal of the coaxial needle stylet [75]. The last case report lacked an adequate ascertainment of exposure and causality between air embolism and PTLB because the patient only had local anesthesia with lidocaine and then presented with symptoms [63].

### Incidence of symptomatic air embolism of PTLB

**Fig. 2:**
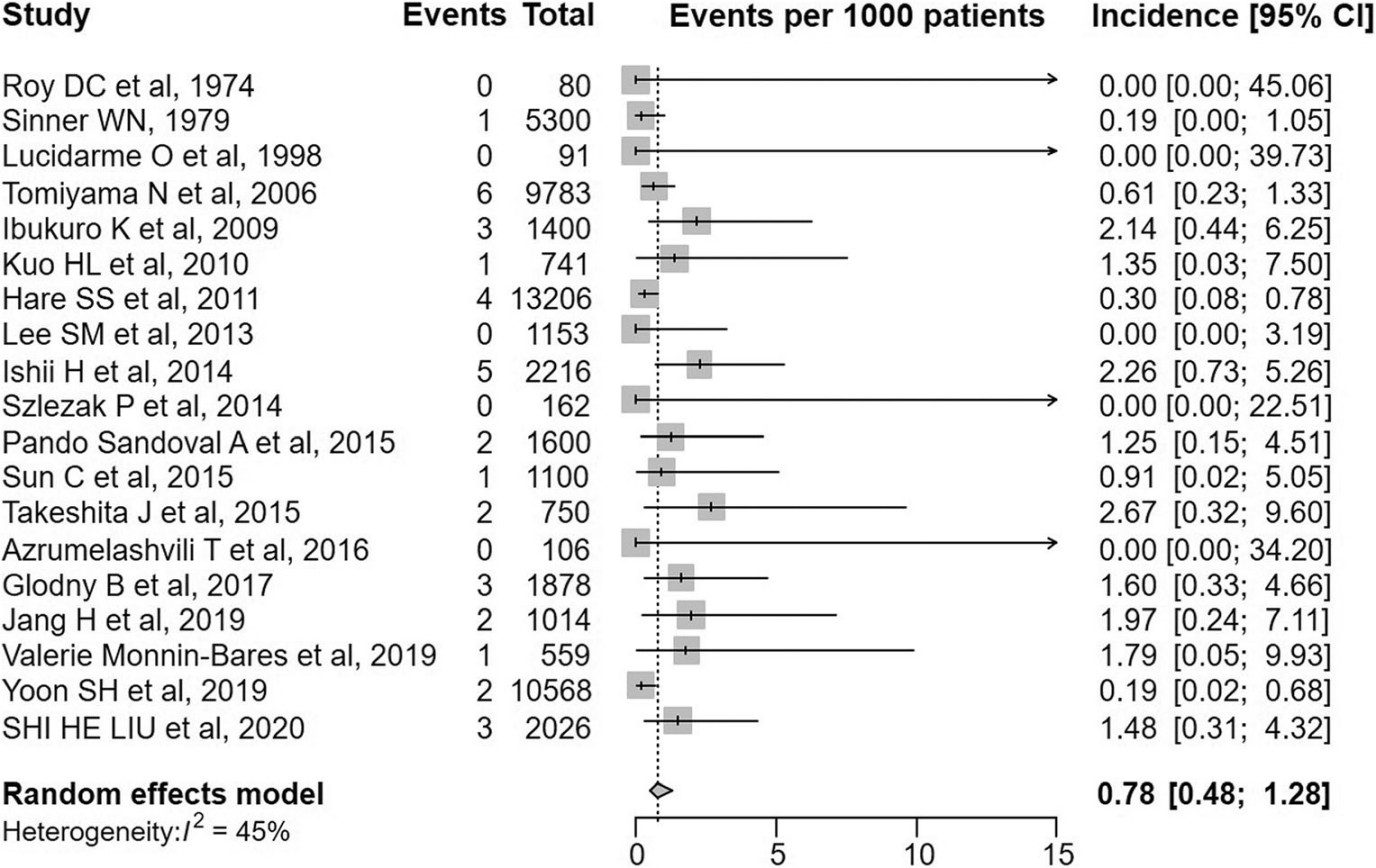
Forest plot for incidence of symptomatic air embolism after percutaneous transthoracic lung biopsy (events per 1000 patients)

**Fig. 3:**
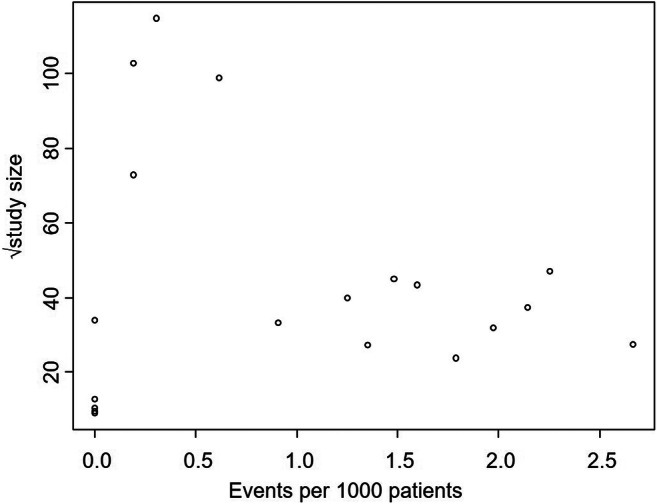
Funnel plot of included studies evaluating incidence of symptomatic air embolism after percutaneous transthoracic lung biopsy (events per 1000 patients)

Nineteen articles were included in the analysis of the incidence of symptomatic air embolism after PTLB, and the pooled incidence was 0.08% (95% confidence interval [CI], 0.048–0.128; *I*^2^ = 45%) (Fig. 2). The subgroup analysis and meta-regression (Table 1) showed that CT guidance (vs. other modalities; *p* = 0.002) and study size < 2500 (vs. ≥ 2500; *p* < 0.001) were significant factors explaining heterogeneity across studies. There was no evidence of publication bias on a visual evaluation of funnel plots (Fig. 3).

**Table 1:** Result of subgroup analysis and meta-regression of incidence of symptomatic air embolism after percutaneous transthoracic lung biopsy

Covariate		Number of studies	Subgroup analysis		Meta-regression		
			Events per 1000 patients [95% confidence interval]*	*I*^2^ (%)	Beta [95% confidence interval]	*p* value	*I*^2^* (%)
Guidance modality	CT guidance	13	1.25 [0.82, 1.89]	11.9	0	0.002	6.9
	Other modalities	6	0.24 [0.11, 0.50]	0	− 1.66 [− 2.62, − 0.7]		
Study size	≥ 2500	4	0.34 [0.19, 0.58]	0	0	< 0.001	0.0
	< 2500	15	1.55 [1.03, 2.33]	0	1.53 [0.8, 2.26]		
Publication year	After 2000	16	0.87 [0.53, 1.43]	45	0	0.171	47.6
	Before 2000	3	0.18 [0.03, 1.30]	0	− 1.71 [− 4.22, 0.81]		

*Percentage of residual heterogeneity unexplained by accounted factors in the model

### Characteristics of individual patient data

Details on the clinical, radiological, and biopsy-related information that could be matched to each individual from the 123 patients described in the 90 eligible articles are presented in Table 2.

**Table 2:** Baseline clinical, radiological and percutaneous transthoracic lung biopsy-related characteristics of 123 patients

**Age (years)**	64.2 ± 11.6 (range, 25–85)	**Distance between pleura and target lesion**	
≥ 65 years	56 (45.5%)	≥ 1 cm	16 (13%)
< 65 years	66 (53.7%)	< 1 cm	8 (6.5%)
Not specified	1 (0.8%)	Not specified	99 (80.5%)
**Sex**		**Biopsy needle gauge**	
Men	84 (68.3%)	≥ 20 gauge	55 (44.7%)
Women	39 (31.7%)	< 20 gauge	52 (42.3%)
**Underlying disease**		Not specified	16 (13%)
Yes	72 (58.5%)	**Coaxial technique**	
No	40 (32.5%)	Yes	68 (55.3%)
Not specified	11 (8.9%)	No	37 (30.1%)
**Mechanical ventilation**		Not specified	18 (14.6%)
Yes	6 (4.9%)	**Cough**	
No	106 (86.2%)	No	74 (60.2%)
Not specified	11 (8.9%)	Yes	45 (36.6%)
**Long diameter of target lesion (cm)**	2.3 ± 1.6 (range, 0.5–11); Median 2	Not specified	4 (3.3%)
≥ 2 cm	42 (34.1%)	**Symptom or sign**	
< 2 cm	40 (32.5%)	Yes	98 (79.7%)
Not specified	41 (33.3%)	No	19 (15.4%)
**Target lesion type**		Not specified	6 (4.9%)
Solid or consolidation	93 (75.6%)	**Cardiac symptom**	
Ground-glass opacity	8 (6.5%)	Yes	32 (26.0%)
Not specified	22 (17.9%)	No (other symptoms)	66 (53.7%)
**Cavitary/cystic change**		Not specified	25 (20.3%)
Yes	17 (13.8%)	**Loss of consciousness**	
No	84 (68.3%)	Yes	42 (34.1%)
Not specified	22 (17.9%)	No (other symptoms)	56 (45.5%)
**Lobar location**		Not specified	25 (20.3%)
RUL, RML, RLL, LUL, LLL, not specified	17 (13.8%), 10 (8.1%), 28 (22.8%), 13 (10.6%), 33 (26.8%), 22 (17.9%)	**Location of air embolism**	
Upper and middle lobe	42 (34.1%)	Coronary arteries or intracranial space	69 (56.1%)
Lower lobe	67 (54.5%)	Others	48 (39%)
Not specified	14 (11.9%)	Not specified	6 (4.9%)
**Guidance modality**		**Hyperbaric oxygen treatment**	
CT	112 (91.1%)	Yes	39 (31.7%)
Others	8 (6.5%)	No	82 (66.7%)
Not specified	3 (2.4%)	Not specified	2 (1.6%)
**Biopsy methods**		**Trendelenburg position**	
Core biopsy	92 (74.8%)	Yes	30 (24.4%)
Aspiration biopsy	27 (22%)	No	80 (65%)
Not specified	4 (3.2%)	Not specified	13 (10.6%)
**Patient position**		**Other complication**	
Supine	31 (25.2%)	Yes	65 (52.8%)
Prone	53 (43.1%)	No	40 (32.5%)
Lateral decubitus	20 (16.3%)	Not specified	18 (14.6%)
Not specified	19 (15.4%)	Outcome*	
**Maximum needle gauge**		Survival without any sequela	89 (72.3%) [66, 67.3%]
≥ 19 gauge	65 (52.8%)	Survival with sequela	12 (9.8%) [11, 11.2%]
< 19 gauge	42 (34.1%)	Death	22 (17.9%) [21, 21.5%]
Not specified	16 (13%)		

*Number and percentage of outcome in patients with symptomatic air embolism (*n* = 98) are suggested in each bracket

Seventy-two of the 123 patients (58.5%) had underlying diseases, of which cancer was the most common (36.1%, 26 of 72), followed by chronic obstructive pulmonary disease (COPD) (23.6%, 17 of 72). Other diseases were asthma, idiopathic pulmonary fibrosis, hypertension, diabetes, and pneumoconiosis.

For 20 patients with lateral decubitus position during the procedure, targeted lungs were non-dependent in 18 patients while 2 patients had unspecified information of whether targeted lung was non-dependent or dependent.

Ninety-eight of the 123 patients (79.7%) had immediate symptoms or signs during or after PTLB. The most common symptom was loss of consciousness (42.9%, 42 of 98), followed by cardiac-related symptoms or signs (32.7%, 32 of 98; chest pain and myocardial infarction) and neurological symptoms or signs (24.5%, 24 of 98; e.g., reduction of sensation, motor weakness, dysarthria, blindness, dysarthria, convulsion). Other symptoms or signs included a change in blood pressure, voiding problems, dyspnea, and hemoptysis.

In terms of the location of the air embolism, 69 patients had air emboli in the coronary arteries or intracranial spaces (59%, 69 of 117). Other reported locations included the aorta (21.4%, 25 of 117), left atrium (17.1%, 20 of 117), left ventricle (19.7%, 23 of 117), right atrium (1.7%, 2 of 117), right ventricle (0.9%, 1 of 117), pulmonary artery (1.7%, 2 of 117), pulmonary vein (5.1%, 6 of 117), mesenteric artery (0.9%, 1 of 117), spinal artery (0.9%, 1 of 117), and the systematic venous system including the superior vena cava and subclavian vein (0.9%, 1 of 117).

In regard to patients’ outcomes, 66 of the 98 patients (67.3%) with symptomatic embolism survived without any sequelae, whereas 11 (11.2%) and 21 (21.5%) patients survived with sequelae and died, respectively. Therefore, 32 of 98 patients (32.7%) with symptomatic embolism were considered to have an unfavorable outcome.

### Risk factors for symptomatic air embolism after PTLB

In the analysis of risk factors for symptomatic air embolism after PTLB, 117 patients were included after the exclusion of 6 patients whose symptoms or signs were not described in the corresponding studies. In the univariate analysis, a younger age than 65 years (*p* = 0.014), aspiration biopsy rather than core biopsy (*p* = 0.04), a ≥ 19-gauge needle (*p* = 0.004), and location of the air embolism in the coronary arteries or intracranial space (*p* < 0.001) showed significant associations with symptomatic air embolism while factors with *p* values between 0.05 and 0.1 were the presence of an underlying disease (*p* = 0.091) and supine position rather than lateral decubitus position (*p* = 0.094) (Table 3). In a multivariate analysis of 93 patients who had clear information on the above characteristics, the presence of an underlying disease (odds ratio [OR] = 5.939; 95% confidence interval [CI] = 1.029–34.279; *p* = 0.046), use of a ≥ 19-gauge needle (OR = 10.046, 95% CI = 1.103–91.469; *p* = 0.041), and location of the air embolism in the coronary arteries or intracranial spaces (OR = 19.871; 95% CI = 2.725–14.925; *p* = 0.003) were found to be significant risk factors. Regarding the underlying disease, cancer, COPD, hypertension, diabetes, idiopathic pulmonary fibrosis, and asthma were associated with symptomatic air embolism in descending order.

**Table 3:** Univariate analysis and multivariate logistic regression analysis to investigate risk factors for symptomatic air embolism after percutaneous transthoracic needle biopsy in 117 patients

	Univariate analysis			Multivariate analysis (*n* = 93)		
	Odds ratio	95% confidence interval	*p* value	Odds ratio	95% confidence interval	*p* value
Age < 65 years (vs. ≥ 65 years)	3.77	1.246, 11.408	*0.014*			
Male (vs. female)	1.73	0.631, 4.745	0.283			
Underlying disease (vs. none)	2.333	0.858, 6.343	*0.091*	5.939	1.029, 34.279	*0.046*
Mechanical ventilation (vs. none)	1.023	0.113, 9.286	1.000			
Diameter of target lesion ≥ 2 (vs. < 2 cm)	0.92	0.312, 2.709	0.879			
Solid lesion (vs. subsolid lesion)	2.888	0.626, 13.326	0.17			
Cavitary/cystic change (vs. none)	1.379	0.279, 6.805	1.000			
Lower lobar location (vs. upper or middle lobe)	1.742	0.626, 4.85	0.285			
CT-guided biopsy (vs. other modality)	1.211	1.111, 1.32	0.587			
Aspiration biopsy (vs. core biopsy)	6.882	0.874, 52.202	*0.04*			
Pleura to target lesion ≥ 1 cm (vs. < 1 cm)	0.9	0.133, 6.08	1.000			
Needle gauge ≥ 19 (vs. < 19)	7.442	1.609, 34.427	*0.004*	10.046	1.103, 91.469	*0.041*
Supine position (vs. prone position)	0.456	0.134, 1.548	0.265			
Supine position (vs. lateral decubitus position)	6.175	0.752, 50.729	*0.094*			
No coaxial technique (vs. use)	0.558	0.197, 1.583	0.269			
Cough (vs. none)	0.797	0.288, 2.205	0.662			
Location of air embolism in coronary arteries or intracranial spaces (vs. other location)	18.371	3.995, 84.482	*< 0.001*	19.871	2.725, 144.925	*0.003*

*p* values of statistically significant variables are in italics

### Prognostic indicators for unfavorable outcomes of air embolism after PTLB

In the analysis of unfavorable outcomes in patients with symptomatic air embolism, aspiration biopsy rather than core biopsy (*p* = 0.013), loss of consciousness as an immediate symptom (*p* = 0.021), and location of the air embolism in the coronary arteries or intracranial spaces (*p* = 0.001) were found to be significant risk factors in the univariate analysis, while factors with *p* values between 0.05 and 0.1 were a younger age than 65 years (*p* = 0.075), lesion size of 2 cm or larger (*p* = 0.093), no use of the coaxial technique (*p* = 0.087), and no application of the Trendelenburg position (*p* = 0.084) (Table 4). Excluding the factors of lesion size and use of the coaxial technique because of the large number of patients with missing data on these factors, a multivariate analysis could be performed of 90 patients. In this analysis, aspiration biopsy rather than core biopsy (OR = 3.302; 95% CI = 1.149–9.492; *p* = 0.027) and location of the air embolism in the coronary arteries or intracranial spaces (OR = 5.173; 95% CI = 1.309–20.447; *p* = 0.019) were significant risk factors for unfavorable outcomes.

**Table 4:** Univariate analysis and multivariate logistic regression analysis to investigate prognostic indicators for unfavorable outcome in 98 patients with symptomatic air embolism after percutaneous transthoracic needle biopsy

	Univariate analysis			Multivariate analysis (*n* = 90)		
	Odds ratio	95% confidence interval	*p* value	Odds ratio	95% confidence interval	*p* value
Age < 65 years (vs. ≥ 65 years)	2.263	0.911, 5.621	*0.075*			
Male (vs. female)	0.889	0.355, 2.227	0.802			
Underlying disease (vs. none)	1.245	0.5, 3.1	0.638			
Mechanical ventilation (vs. none)	3.214	0.508, 20.332	0.329			
Diameter of target lesion ≥ 2 (vs. < 2 cm)	2.779	0.823, 9.379	*0.093*			
Solid lesion (vs. subsolid lesion)	0.765	0.12, 4.866	1.000			
Cavitary/cystic change (vs. none)	1.725	0.529, 5.622	0.362			
Lower lobar location (vs. upper or middle lobe)	0.636	0.245, 1.653	0.352			
CT-guided biopsy (vs. other modality)	0.951	0.165, 5.494	0.955			
Aspiration biopsy (vs. core biopsy)	3.25	1.255, 8.414	*0.013*	3.302	1.149, 9.492	*0.027*
Pleura to target lesion ≥ 1 cm (vs. < 1 cm)	1.0	0.141, 7.099	1.000			
Needle gauge ≥ 19 (vs. < 19)	1.391	0.548, 3.528	0.486			
Supine position (vs. prone position)	0.715	0.261, 1.96	0.514			
Supine position (vs. lateral decubitus position)	1.63	0.499, 5.324	0.417			
No coaxial technique (vs. use)	2.357	0.872, 6.374	*0.087*			
Cough (vs. none)	1.644	0.692, 3.91	0.259			
Cardiac symptom (vs. no cardiac symptom)	2.074	0.857, 5.02	0.103			
Loss of consciousness (vs. no loss of consciousness)	2.732	1.147, 6.511	*0.021*	2.512	0.903, 6.985	0.078
Location of air embolism in coronary arteries or intracranial spaces (vs. other location)	7.123	1.971, 25.747	*0.001*	5.173	1.309, 20.447	*0.019*
Hyperbaric oxygen treatment (vs. none)	1.574	0.653, 3.792	0.311			
No Trendelenburg position (vs. application)	2.571	0.862, 7.669	*0.084*			
Other complication	1.143	0.472, 2.767	0.767			

*p* values of statistically significant variables are in italics

## Discussion

The commonly accepted incidence of air embolism, focusing on symptomatic patients, has been reported to be 0.02 to 0.07% [12–14], and our finding of a pooled incidence of symptomatic air embolism after PTLB of 0.08% is similar to the incidence reported in previous studies. However, some recent studies have reported that the incidence of air embolism after PTLB ranged from 0.21 to 3.8% based on the inclusion of asymptomatic patients with extensive surveys of post-biopsy CT examinations [6, 8, 9, 11, 19, 24]. Because of the major difference between the incidence of asymptomatic and symptomatic embolism unveiled in prior studies, we only pooled studies dealing with symptomatic air embolism.

In the subgroup analysis and meta-regression analysis, studies that only analyzed CT-guided PTLB had significantly more events than studies using other modalities (e.g., fluoroscopy and ultrasonography). The finding is unsurprising because air embolism could be detected directly with post-biopsy CT surveillance in studies of CT-guided PTLB. To support this inference, the majority of cases in those studies included cases of air embolism located in the heart, coronary arteries, aorta, and pulmonary vein, which are in the scan range of chest CT. Study size was another significant factor contributed to the heterogeneity, as the average incidence in studies that enrolled a small number of patients was higher than that of large-scale studies. It is speculated that when air embolism occurs after PTLB in a small-scale study, its incidence may be estimated as much higher than would be the case in a large-scale study.

Since prompt recognition of air embolism is critical for optimizing patients’ clinical course [6, 18], it is paramount to investigate the risk factors of symptomatic air embolism. We found the presence of an underlying disease, larger gauge of the biopsy needle, and location of the air embolism in the coronary arteries or intracranial spaces were predictive factors for symptomatic air embolism. COPD was the most common underlying disease (23.6% of the included patients), which may be explained by the fact that COPD can result in extended exposure of the pulmonary vessel lumen to the airway, thereby increasing the chance of air bubbles entering the bloodstream [56]. The relationship of needle gauge with air embolism after PTLB is controversial, because a number of cases of air embolism occurred even when a small needle was used [8, 72, 77, 78]. To explain the association found in our study, it is speculated that once an air embolism occurs, the use of a large needle results in higher chance of a sufficiently large air bolus entering the bloodstream and causing symptoms [9]. Finally, the presence of an air embolism in the coronary or cerebral arteries was linked to a severely deteriorated prognosis, with outcomes including myocardial infarction and permanent neurological deficits [6, 61, 85, 111]. Concordant with prior studies, location of the air embolism in the coronary arteries or intracranial spaces was a significant predictor of symptomatic air embolism.

Of note, we found that 32.7% (32 of 98) of symptomatic patients with air embolism had unfavorable outcomes. Of those 32 patients, 21 died and 11 survived, but had neurological sequelae. Our results suggest that two-thirds of patients with symptoms or signs eventually survive without any sequelae. The use of aspiration biopsy rather than core biopsy and the presence of the air embolism in coronary arteries or intracranial spaces were significant prognostic indicators for unfavorable outcomes in our study. We suggest that aspiration biopsy may cause a large air bolus due to the back-and-forth technique inherently involved in this procedure, which results in a high likelihood of air entering the bloodstream. As was described above, the organ involved in air embolism is a critical factor for patients’ symptoms and outcomes, and it is therefore natural for location of the air embolism in the coronary arteries and intracranial spaces to be a significant predictor of unfavorable outcomes.

There has been controversy about application of the Trendelenburg position for patients with air embolism [61, 90, 111, 113–115]. According to some authorities, the Trendelenburg position is only appropriate for cases of venous air embolism, as it keeps the air bolus in the right ventricular cavity [114, 115]. Nevertheless, our systematic review revealed that patients who had been placed in the Trendelenburg position for air embolism tended to have favorable outcomes (20 of 25 patients, *p* = 0.084 in the univariate analysis) and most of these patients had arterial air embolism (23 of 25 patients). Although application of the Trendelenburg position did not show significance in the multivariate analysis, this tendency of the Trendelenburg position to be associated with favorable outcomes should be investigated in further studies.

Two main limitations should be stated. Most importantly, this study comprised mostly case reports or series due to the rarity of air embolism after PTLB, and therefore, the certainty of the evidence could be low [20]. For instance, hyperbaric oxygen therapy is believed to be a mainstay treatment for air embolism because it helps reduce bubble volume, biochemical reactions at the blood-gas interface that result in hemostasis, and endothelial damage [69, 79, 90]. Despite its proven usefulness, it was not associated with patients’ outcomes in our study. Second, we did not receive clarifications on missing or unreported data in several cases. Consequentially, relatively few patients were included in the analysis of risk factors, which might have caused potential bias and limited our results.

In conclusion, the pooled incidence of symptomatic air embolism after PTLB was 0.08%, and one-third of cases had sequelae or died. Symptomatic air embolism was more frequent in patients with an underlying disease (*p* = 0.046), with the use of a ≥ 19-gauge needle (*p* = 0.041), and when the air embolism was located in the coronary arteries or intracranial spaces (*p* = 0.003). The use of aspiration biopsy rather than core biopsy (*p* = 0.027) and location of the air embolism in the coronary arteries or intracranial spaces (*p* = 0.019) were independent prognostic indicators of unfavorable outcomes.

## Abbreviations

COPD: Chronic obstructive pulmonary disease
PTLB: Percutaneous transthoracic lung biopsy
